# More about the Association of Faculties

**Published:** 1899-12

**Authors:** L. A. Merillat

**Affiliations:** Secretary-Treasurer


### MORE ABOUT THE ASSOCIATION OF FACULTIES.

EDITOR OF THE JOURNAL OF COMPARATIVE MEDICINE, ETC.:

"What has become of the Association of Faculties?" asks Prof. Dr. Schwartzkopf, in a recent number of the *American Veterinary Review*. I have a guilty suspicion that the question was aimed at the officers of that organization, which, by the way, is now called the Association of Veterinary Faculties and Examining Boards of North America. If not, its answer at least should emanate from them. If the question had been, "Why was there no meeting in 1899?" I might readily have answered, "Because there were no officers present to call such a meeting, and especially because I, the Secretary-Treasurer, neglected to send the minutes, programmes, account books, etc., to the place of meeting." To be more explicit, I might express myself in the language of General White in his report of the famous mule stampede at the Battle of Dundee, "I alone am responsible." And I beg to assure the members of the Association that I use the expression with the same sincerity and earnestness as the General did. But, on the other hand, if the Association is entirely lost, as the query implies, then, in justice to myself, I shall agree to share only a part of the responsibility.

At the Omaha meeting Dr. Stalker and myself were intrusted with the two executive offices, and, owing to the painful fact that neither of us "showed our faces" at the New York meeting, we clearly deserve the severest censure. We stand indicted for gross malfeasance, and (I cannot plead for Stalker) plead guilty, guilty, guilty.

I worked with some earnestness during the summer at the difficult task of securing a literary programme for the New York meeting, and I received encouraging replies from such faithful workers as Law, Harger, Williams, Pearson, Robinson and many others. All were of the opinion, in regard to the future of the Society, that its original aim could never be consummated, and that the next few meetings at least should be devoted to the amelioration of teachers as individuals instead of that of the colleges as a whole—that is to say, the sentiment of transforming the Society into a teachers' institute was freely expressed. I found t impossible to attend the meeting—a circumstance I very much regret—but I expected to send the Association's property to New York with Dr. Stalker when he passed through Chicago en route to the meeting. Although he had once during the summer expressed some doubt as to his ability to attend, I did not for one moment doubt that he would not be there to preside. In this I erred, and when the error was realized it was too late to send the books.

This circumstance should not, however, materially affect the future of the institution, and if it has received its death-blow from this circumstance the prognosis must have been unfavorable any way. I trust I am not too optimistic when I see in the masterly address of Law, the scholarly essay of Harger, the lucid, instructive discussion of Williams, and the paper of Clement on State Examinations, the foundation of a useful and progressive veterinary teachers' institute. (See An. Rept. A. V. M. A., 1899.) Let us hope that such an organization will be born of the obsolete one. So far as the "Examining Board Contingent" is concerned, there need be no impediment, as the relation between the teacher and examiner must always be a close one.

Unless lawfully impeached, I shall act under the constitutional clause that compels officers to retain their seats until their successors are duly inaugurated. Discussions through these columns are solicited.

L. A. MERILLAT,
Secretary-Treasurer.

